# Investigating the spatio-temporal variation of hepatitis A in Korea using a Bayesian model

**DOI:** 10.3389/fpubh.2022.1085077

**Published:** 2023-01-20

**Authors:** Jaehong Jeong, Mijeong Kim, Jungsoon Choi

**Affiliations:** ^1^Department of Mathematics, Hanyang University, Seoul, Republic of Korea; ^2^Research Institute for Natural Sciences, Hanyang University, Seoul, Republic of Korea; ^3^Department of Statistics, Ewha Womans University, Seoul, Republic of Korea

**Keywords:** hepatitis A virus, spatio-temporal analysis, spatio-temporal models, zero-inflated Poisson, Bayesian hierarchical modeling, Korea

## Abstract

Hepatitis A is a water-borne infectious disease that frequently occurs in unsanitary environments. However, paradoxically, those who have spent their infancy in a sanitary environment are more susceptible to hepatitis A because they do not have the opportunity to acquire natural immunity. In Korea, hepatitis A is prevalent because of the distribution of uncooked seafood, especially during hot and humid summers. In general, the transmission of hepatitis A is known to be dynamically affected by socioeconomic, environmental, and weather-related factors and is heterogeneous in time and space. In this study, we aimed to investigate the spatio-temporal variation of hepatitis A and the effects of socioeconomic and weather-related factors in Korea using a flexible spatio-temporal model. We propose a Bayesian Poisson regression model coupled with spatio-temporal variability to estimate the effects of risk factors. We used weekly hepatitis A incidence data across 250 districts in Korea from 2016 to 2019. We found spatial and temporal autocorrelations of hepatitis A indicating that the spatial distribution of hepatitis A varied dynamically over time. From the estimation results, we noticed that the districts with large proportions of males and foreigners correspond to higher incidences. The average temperature was positively correlated with the incidence, which is in agreement with other studies showing that the incidences in Korea are noticeable in spring and summer due to the increased outdoor activity and intake of stale seafood. To the best of our knowledge, this study is the first to suggest a spatio-temporal model for hepatitis A across the entirety of Korean. The proposed model could be useful for predicting, preventing, and controlling the spread of hepatitis A.

## 1. Introduction

Unlike Hepatitis B or C, Hepatitis A virus (HAV) is not transmitted through blood, but by consuming food or water contaminated with HAV or by contact with an infected person (1). World Health Organization (1) reported that the number of deaths from HAV in 2015 was estimated to be about 11,000 worldwide, accounting for 0.8% of deaths from viral infections. A case-control study of the HAV outbreak in Shanghai in 1988 supported that clams were a carrier of the virus (2). In Korea, HAV is often reported to spread through shellfish consumption (3). In India, a case-control study showed a high association between pipe water contamination and HAV infection (4). It particularly occurs in underdeveloped areas where personal hygiene management is poor, and HAV infection cases are decreasing in countries with an improvement in socioeconomic level, clean water management system, and HAV vaccination (5). However, the incidence rate has recently increased rapidly, in young adults who grew up in a hygienic environment in Korea (6).

Several studies have investigated the effects of socioeconomic and epidemiological factors such as age, medical level, and hygiene level on HAV in various countries. For example, it was noted that a significantly lower rate of HAV infection in people coupled with moderate to high socioeconomic conditions in Brazil, Argentina, and Mexico in Tapia-Conyer et al. (7). Mantovani et al. (8) discovered that a region with a high incidence of HAV had a weak socioeconomic condition in Brazil, thus emphasizing the need for hygiene improvement and better water treatment in the western Brazilian Amazon to reduce infectious disease outbreaks. Dogru et al. (9) classified children under the age of 15 in Turkey into three categories and analyzed the spatial patterns of HAV occurrence. In Turkey, the incidence of HAV was reported to be high in areas where water and sewage facilities are not well equipped. Copado-Villagrana et al. (10) pointed out that HAV infections were mainly found in the metropolitan areas of southern and western Mexico, noting that it may be associated with poor medical services in the most marginalized areas. Zheng et al. (11) characterized changes in the incidence and mortality of HAV in various age groups and regions in China from 1990 to 2018 and evaluated the effectiveness of the nationwide expanded program on immunization. The spread of the disease was decreased by expanding vaccinations and improving hygiene facilities. Shanmugam et al. (12) showed that primary infection of HAV among the older age group in India has recently decreased with improved living conditions.

Weather-related variables are associated with the incidence of HAV. According to Cann et al. (13), in extreme weather conditions such as hurricanes, cross-contamination of water supply and sewage may affect the transmission of waterborne diseases. In Brazil, cases of HAV infection increased during the rainy season (14). In state of Pará, Brazil, monthly accumulated precipitation was positively correlated with the incidence of HAV (15). Tosepu (16) found a strong relationship between HAV and weather change, particularly rainfall and floods, in several areas, such as Spain, India, China, and Egypt. Baek et al. (17) showed that weekly precipitation and maximum temperature tended to decrease the incidence rate ratio of HAV in Seoul, Korea from the analysis of time series with past 1–6 week lags. In Seoul, the capital of South Korea, nearly 100% of households receive sterilized tap water and most citizens drink purified or clean spring water. In addition, since Seoul is geographically less affected by typhoons and floods, HAV is not likely to be transmitted due to cross-contamination of water supply and sewerage due to heavy seasonal rains as in other regions (17). In this respect, HAV incidence seems to be related to weather conditions, but the pattern may differ across regions. Fares (18) pointed out that some specific months are associated with a higher incidence of HAV in most countries around the world, but the exact reason for the seasonality of HAV has yet to be known. Several researchers have suggested climatic and behavioral factors such as swimming and traveling may play an important role in seasonal disease incidence (18). Moon et al. (19), based on the dataset in Korea from 2011 to 2013, reported that most HAV cases occurred from March to June. People have more outdoor activities as the weather becomes warmer during this period; thus, they are at risk of being exposed to tainted drinking water and uncooked seafood. Both are well-known risk factors for HAV infections (20). Thus, weather-related variables such as temperature and precipitation should be considered when studying HAV occurrences because weather conditions can affect people's behavior.

Various spatio-temporal analyses have been conducted to explore and understand the risk of HAV in terms of spatial and temporal structures. Gomez-Barroso et al. (21) analyzed the space-time risk of HAV using standardized incidence ratios (SIR; the ratio between actual and expected cases) and the posterior probability of the smoothed relative risk (RR; the ratio of the outcome probability for the exposed group to the probability for the unexposed group) in Spain at the municipal level from 1997 to 2007. Stoitsova et al. (22) applied the Global Moran's I index for spatial autocorrelation across Bulgaria concerning the risk of HAV infection and SIR across the nation for the whole period from 2003 to 2013 and two divided periods (2003–2008 and 2008–2013). Leal et al. (23) explored the spatio-temporal patterns of HAV outbreaks before (2008–2013) and after (2014–2017) the implementation of the national public immunization program in Pará State, a region of Brazil with severe endemic disease. Space-time scan statistics were applied to detect spatio-temporal clusters. Moreover, Leal et al. (15) investigated the association between environmental and socio-demographic data in HAV transmission in Pará State, Brazil, using various models, including generalized linear models, multilayer perceptron (MPL) deep-learning algorithm, gradient boost, decision tree, and histogram gradient boost (HGB). To reflect the spatial variation, the longitude and latitude of each municipality were used as covariates in the model.

As discussed above, HAV is related to many factors, such as socioeconomic and epidemiological factors, weather-related factors, and spatio-temporal variations; therefore, referring to the status of diseases in other countries is not enough. For a better understanding of the HAV of the country, we should consider not only its local and national characteristics but also its social and hygienic situation.

With rapid urbanization, Korea has become cleaner. Since the first sewage treatment plant was established in Korea in 1976 (24), the number of sewage treatment plants has gradually increased, and accordingly, the number of HAV infections caused by contaminated water has decreased rapidly. As of 2020, the water supply rate is 97% nationwide, 100% in Seoul and 45.7% in Cheongyang-gun, Chungcheongnam-do. In the same year, the sewage supply rate was 94.5% nationwide, 100% in Seoul, and 5.4% in Ulleung-gun, Gyeongsangbuk-do. Although Korea's large cities have well-equipped water and sewage facilities, some areas of rural and fishing villages are in poor condition. Children with acute HAV are asymptomatic or mildly symptomatic, and antibodies (IgG anti-HAV) develop, resulting in lifelong immunity (6). According to Yoon et al. (6), IgG anti-HAV seropositivity in the Korean young adult population was low and a clean environment may lead to a decrease in the natural immune system population. Since the HAV vaccination began in 1997, the vaccination rate for 3-year-old infants in Korea exceeded 95% in 2019, according to the Korea Disease Control and Prevention Agency. People without antibodies are at an increased risk of exposure to HAV during active adolescence because they do not contract HAV as a child. When infected as an adult, the immune response is severe and symptoms such as jaundice appear; in severe cases, acute liver failure can lead to death (6). People born after 1980, when the environment was cleaner and the HAV vaccine was yet developed, have been reported to be more susceptible to HAV (25, 26). Other similar studies on the seroprevalence of HAV antibodies have been reported steadily over time (27–30).

Some efforts have been made to investigate and understand the status of HAV in Korea using statistical approaches. Research on the frequency analysis of the number of HAV infections by year, region, and age have been steadily reported (19, 31–33). Moon et al. (19) studied the epidemiological status of HAV cases in Korea between 2011 and 2013. They described significant differences in the incidence of HAV between months, regions, sexes, and age groups. In particular, they classified regions into five clusters according to the RR. Using Moran's I and scan statistics, they found clear and existing regional differences in the incidence of HAV; however, their approach was limited to exploratory data analysis. In addition, RR does not always show correct risks (34). Seo et al. (25) studied the effect of socioeconomic status and environmental hygiene by region on the incidence of HAV based on the registered national population of Korea and national health insurance data from 2004 to 2008 using a Poisson regression model. Choi (35) conducted spatial hotspot detection of monthly incidences of HAV using spatial scan statistics and investigated the effects of socioeconomic factors using the Bayesian spatial Poisson regression model. Even though the HAV data were monthly, the proposed spatial model considered yearly incidence data and was independently applied. Thus, this study did not consider temporal and spatial variations for consecutive periods. Choi (36) analyzed HAV incidence data from 2007 to 2012 in Korea using a Bayesian spatio-temporal Poisson regression model. In Korea, HAV occurs more in summer and less in winter; therefore, seasonal factors are reflected in the model using a sine/cosine function. However, this study did not consider socioeconomic or weather factors. Baek et al. (17) conducted a time series analysis to explain the influence of factors, such as temperature and precipitation, on the incidence of HAV in Seoul, Korea. By minimizing the influence of other factors and limiting the study region to a place with a similar lifestyle, they could explain the association between weather-related factors and HAV incidence. However, it is questionable whether the same result can hold for other regions rather than Seoul in Korea. Also, further investigation of the association between HAV cases and variables other than weather is required.

In this study, we aimed to investigate the spatio-temporal variation of HAV in Korea and effects of socioeconomic and weather-related factors using a flexible spatio-temporal model. We proposed a Bayesian spatio-temporal zero-inflated Poisson regression model of weekly HAV incidence in Korea to estimate the effects of risk factors. This study is the first to develop a spatio-temporal model of HAV incidence across the entirety of Korean, with various socioeconomic factors. The advantage of this study is that the proposed model could be useful in predicting, preventing, and controlling the spread of hepatitis A.

## 2. Materials and methods

### 2.1. Description of data

We considered HAV cases as a response variable and socioeconomic, environmental, and weather-related factors as explanatory variables. The dataset for 250 nationwide districts from 2016 to 2019 was obtained from the Korea Disease Control and Prevention Agency. The weekly HAV cases at the district level (called si/gun/gu) had many zero counts (73.5%), suggesting zero-inflated statistical modeling.

We considered income, education level, and fertility rate as socioeconomic factors, because they affect the quality of life. The income variable is defined as the average monthly income per person at the district level. We calculated the high education rate as the proportion of educational attainment of a university degree or higher among the population in their 20s or an older age. The fertility rate was obtained from the actual fertility rate of women 15–49 years in Statistics Korea. We also considered the male proportion because people with active social activities are more likely to be exposed to HAV, and previous studies, including Moon et al. (19), found that the infection occurred more often among men than that among women. Since people born around the 1980s in Korea have a weak tendency toward HAV immunity (26), we considered the age group of 30–49 years. Jacobsen (20) mentioned that diverse epidemiological profiles should be treated as risk factors for HAV, and the number of registered foreigners was used for analyzes. Based on the result of Choi (35), we included the number of medical doctors per 1,000 people as a factor.

For environmental factors, we considered the water supply and sewage treatment facility rates obtained annually from Statistics Korea. Water supply plays an important role not only in terms of health and sanitation but also in industry and firefighting. Waterworks are essential for daily life, but the water system capacity varies depending on several conditions, such as the residential environment (including the housing structure) and the financial status of the local government. Therefore, the water supply rate can be used to evaluate the quality of the local living environment. According to statistics published by the Ministry of Environment of Korea, the water supply rate in Korea increased from 80.1% in 1991 to 97.3% in 2019 due to the government's continuous infrastructure expansion. However, the water supply rate varied between regions. For example, Seoul and Daegu reached 100%, but Jeju Island did not even reach 90%. The gap between the city and rural (and small town) areas is sufficiently large to be ignored. The public sewage treatment facility rate for the population is the ratio of the population beneficiaries of public sewerage services, and the closer it is to 100%, the higher the ratio of the population beneficiaries of public sewage services.

The three weather variables of interest, average temperature, total precipitation, and average humidity, were obtained from the Korea Meteorological Administration. The weather datasets were measured using two systems, an automatic weather system (AWS) and automated synoptic observing system (ASOS), at distinct weather stations (up to 510 and 103 stations, respectively). These measured values should be located in the same spatial domain as other factors; thus, we need to fit a surface to irregularly spaced weather values. Here, we combined the datasets from the two systems and subsequently predicted the weather values at each time and location of interest. To achieve this goal, we used the Kriging model based on a Gaussian spatial random process with a Matérn covariance function at a given time (37).

We report a list of factors for this study in Table 1 and their data sources in Supplementary Table S1. Here, HAV cases and weather-related factors were measured weekly, but socioeconomic and environmental factors were collected yearly. For the socioeconomic and environmental factors, we obtained datasets from different statistics from various institutions. However, we can conveniently access all statistics through the Korean Statistical Information Service (KOSIS).

**Table 1:** Description of data set (outcome, socioeconomic factors, environmental factors, and weather-related factors).

**Variable type**	**Variable**	**Time unit**
Outcome	HAV cases	Weekly
Socioeconomic factor	Total income per person (1 million won)	Yearly
	High education rate (%)	2015
	Total fertility rate per woman aged 15–49 years	Yearly
	Proportion of males (%)	Yearly
	Proportion of people aged 30–49 years (%)	Yearly
	Log(number of foreigners)	Yearly
	Number of doctors per thousand people	Yearly
Environmental factor	Water supply rate (%)	Yearly
	Sewage treatment facility rate (%)	Yearly
Weather-related factor	Average temperature (°C)	Weekly
	Total precipitation (mm)	Weekly
	Average humidity (%)	Weekly

### 2.2. Spatial and temporal association measures

First, weekly HAV cases in a given district are now becoming time series data. Thus, we considered the autocorrelation function (ACF) and partial autocorrelation function (PACF) to examine the temporal association. The ACF corresponds to the correlation between the time series with a lagged version of itself, whereas the PACF measures the additional correlation explained by each successive lagged term. Although these two functions are slightly different, they are both measures of the association between current and past series values. For more details on the ACF and PACF, we refer to Brockwell and Davis (38).

In contrast, HAV cases in districts given at a time are areal data. In this case, we used Moran's I index measuring the strength of spatial associations among districts (39) to examine the spatial association. It is a spatial analog of the measure of association in a time series. In addition, Moran's I index explores a specific type of spatial clustering (40). The proximity matrix consists of weights that spatially connect two districts in a certain manner. Here, two districts closer to one another have more weight than those farther away. Moran's I index coupled with the proximity matrix can be interpreted as follows: a negative value corresponds to some clustering of dissimilar values, a zero value corresponds to perfect randomness, and a positive value indicates some clustering of similar values.

All the analyzes were performed using R software (version 4.1.0; https://www.r-project.org). We used “ape” (41) and “fields” (42) packages to compute the distances among districts and the global Moran's I index.

### 2.3. Statistical model

A Bayesian space-time regression model was developed to investigate the association between socioeconomic, environmental, and weather-related factors and HAV cases and to account for the space-time-dependent structures in the data. As there were many zero values in the weekly district-level HAV cases data, we used a zero-inflated Poisson (ZIP) distribution. Moreover, we considered the two-stage framework proposed by Lawson et al. (43) to overcome the spatial confounding bias problem.

In the first stage, the number of cases for district area *s* (= 1, 2, ⋯ , *S*) and weekly time index *t* (= 1, 2, ⋯ , *T*), *y*_*s,t*_, follows a zero-inflated Poisson distribution as follows:


$$\begin{array}{l} y_{s,t}\sim \text{ZIP}\left(p_{s,t},{\text{λ}}_{s,t}\right), \end{array}$$

where *p*_*s,t*_ is the probability of structural zeros and λ_*s, t*_ is the mean term of the Poisson distribution without structural zeros. The hierarchical structure of the ZIP model can be expressed as


$$\begin{array}{l} \;y_{s,t}|z_{s,t}\sim \text{Poisson}(\mu_{s,t}={\text{λ}}_{s,t}(1-z_{s,t})),\\ \;z_{s,t}\sim \text{Bernoulli}(p_{s,t}),\\ \text{logit}(p_{s,t})=\gamma_{0}+X_{s,j(t)}^{T}\gamma +W_{s,t}^{T}\delta , \end{array}$$

where *j*(*t*) is the yearly time index for socioeconomic and environmental factors and *z*_*s,t*_ is a binary variable with probability *p*_*s,t*_, representing whether it is a structural zero or not. The logit(*p*_*s,t*_) is the linear combination of the intercept γ_0_, and the fixed socioeconomic and environmental factors **X**_*s,j*(*t*)_ and weather-related factors **W**_*s,t*_ with the corresponding coefficient vectors **γ** and ***δ***, respectively. The log RR, log(λ_*s,t*_), is modeled using fixed factors with the corresponding coefficient vectors ***β*** and ***α***.


(1) $$\log({\text{λ}}_{s,t})=\beta_{0}+X_{s,j(t)}^{T}\beta +W_{s,t}^{T}\alpha +\log(N_{s,j(t)}),$$

where β_0_ is the intercept and *N*_*s, j*(*t*)_ is the population density as the off-set. The model of the first-stage only considers fixed factors without spatio-temporal variations.

After fitting the first-stage model using a Bayesian approach, the estimates ${\hat{\mu }}_{s,t}$ were computed using the posterior means. Continuous-type residuals were calculated as follows:


$$\begin{array}{l} {\hat{r}}_{s,t}=\log\left(y_{s,t}+0.1\right)-\log\left({\hat{\mu }}_{s,t}\right), \end{array}$$

where an extra value of 0.1 is added in the residual calculation because of the zero values of *y*_*s,t*_.

In the second-stage, the residuals are modeled to explain the spatio-temporal variations over the first-stage covariates-only model.


(2) $$\begin{array}{l} \;\begin{array}{lll} {\hat{r}}_{s,t} & \sim & \text{N}(ST_{s,t},\sigma_{r}^{2}), \end{array}\\ \begin{array}{lll} ST_{s,t} & = & r_{0}+u_{s}+v_{s}+\eta_{t}+\tau_{t}, \end{array} \end{array}$$

where *r*_0_ ~ N(0, 100) is the intercept. The spatial random component $u_{s}\sim \text{N}\left(0,\sigma_{u}^{2}\right)$ explains the spatially uncorrelated structures, and *v*_*s*_ explains the spatially-correlated structures with conditional intrinsic auto-regressive (CIAR) distribution from Besag et al. (44), $v_{s}\sim \text{CIAR}\left(\sigma_{v}^{2}\right)$. The random components $\eta_{t}\sim \text{N}\left(0,\sigma_{\eta }^{2}\right)$ and $\tau_{t}\sim \text{N}\left(\tau_{t-1},\sigma_{\tau }^{2}\right)$ explain the temporal-uncorrelated and temporal-correlated structures, respectively. After fitting the residual model with a Bayesian approach, the estimated means of the spatio-temporal structures, ${\hat{ST}}_{s,t}$, were obtained. We incorporated the estimated spatio-temporal variations into the fixed covariates. The final model is expressed as follows:


(3) $$\log({\text{λ}}_{s,t})=\beta_{0}+X_{s,j(t)}^{T}\beta +W_{s,t}^{T}\alpha +\log(N_{s,j(t)})+{\hat{ST}}_{s,t}+\epsilon_{s,t},$$

where $\epsilon_{s,t}\sim \text{N}\left(0,\sigma_{\epsilon }^{2}\right)$ is the uncorrelated space-time random component that is not explained by the estimated space-time structure. Finally, the restricted ZIP regression model was fitted using a Bayesian approach to obtain the final estimates for ***β*** and ***α***.

For the parameter estimation, we use non-informative priors, Normal(0, 100), for the coefficient parameters β_0_, γ_0_, ***β***, ***α***, ***γ***, and ***δ***. The standard deviations σ_*u*_, σ_*v*_, σ_η_, and σ_τ_ are assigned to a uniform distribution, Uniform (0, 10). The NIMBLE package developed by de Valpine et al. (45) in the statistical software R was used to produce posterior samples. After discarding samples as a burn-in, 5,000 posterior samples with thin 50 were collected. Codes for the models can be found at https://github.com/JungsoonChoi/STmodeling_HepA.git.

## 3. Results

Table 2 presents a summary of the statistical analysis of all the variables. The proportion of men had a 1.49% interquartile range (IQR = Q3-Q1), and the proportion of people aged 30–49 years had an IQR of 8.63%. Among the socioeconomic factors, these variables showed relatively smaller variations over districts and years. The average temperature and total precipitation had larger standard deviation (SD) values than that of the mean values. Water supply and sewage treatment facility rates have high mean values of 81.05 and 84.69, respectively. Supplementary Figure S1 shows the spatial variation of the average socioeconomic and environmental factors for 2016–2019. Supplementary Table S2 presents the number of HAV cases per 1,000 people divided into five groups for each socioeconomic and environmental factor. For the level of high education, the average number of HAV cases per 1,000 people 0.470 in the G1 districts and 0.615 in the G5 districts, respectively.

**Table 2:** Descriptive statistical analysis.

**Variable**	**Mean**	**SD**	**Q1**	**Q3**	**IQR**
Total income per person	33.68	6.25	29.45	36.40	6.95
High education rate	31.12	11.19	21.70	38.12	16.42
Total fertility rate	1.14	0.27	0.95	1.30	0.35
Proportion of males	50.09	1.29	49.26	50.75	1.49
Proportion of people aged 30–49 years	27.66	5.22	23.14	31.77	8.63
Log(number of foreigners)	7.83	1.22	6.88	8.68	1.80
Number of doctors per thousand people	2.69	2.25	1.70	2.80	1.10
Water supply rate	81.05	30.39	78.97	99.90	20.93
Sewage treatment facility rate	84.69	17.25	74.18	99.50	25.32
Average temperature	12.82	9.69	4.32	21.05	16.73
Total precipitation	21.45	38.02	0.39	24.77	24.38
Average humidity	68.46	10.63	61.01	76.66	15.65

### 3.1. Spatial and temporal distributions of HAV

**Figure 1:**
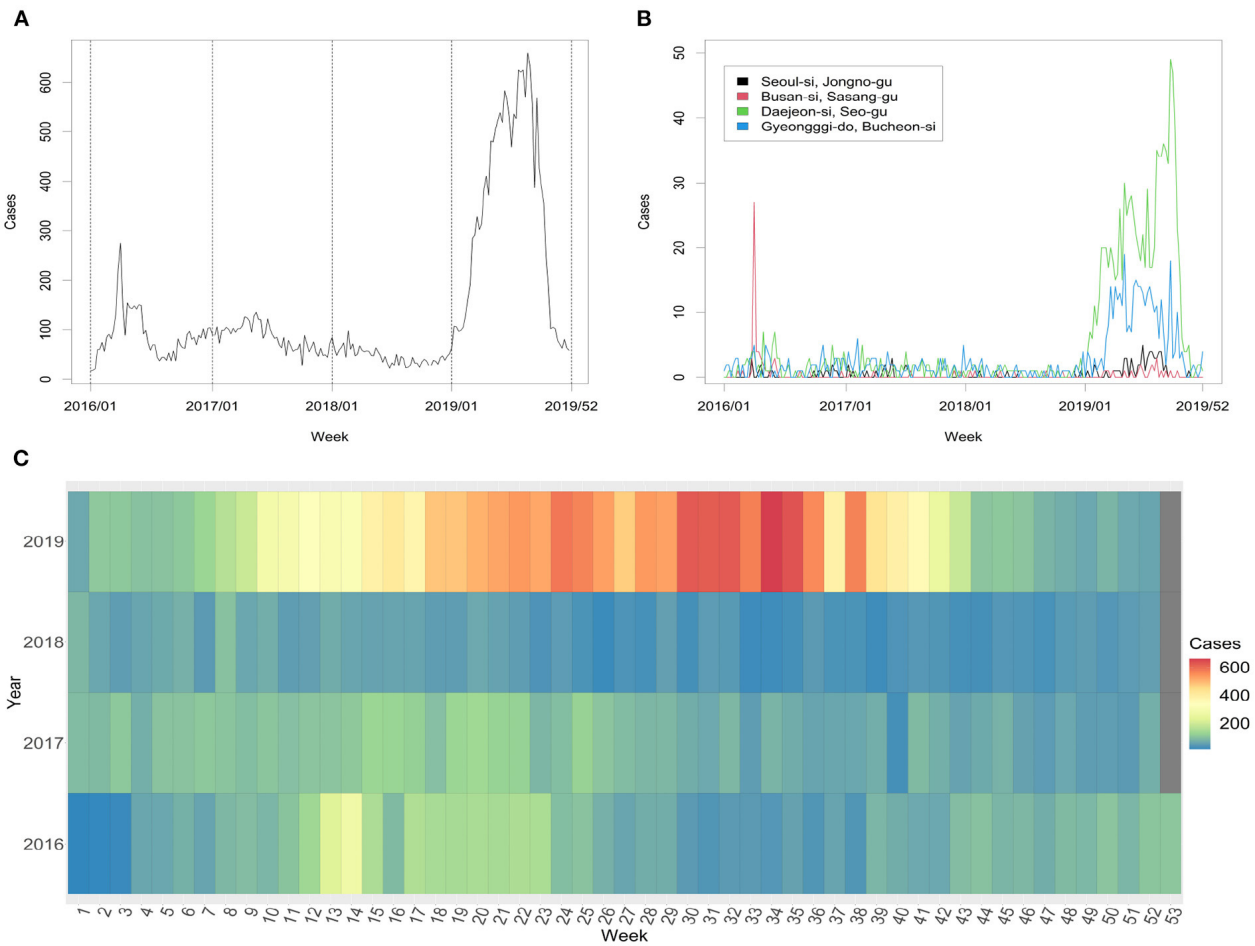
**(A)** Number of HAV cases from 2016 to 2019. **(B)** Weekly HAV cases at selected districts. **(C)** Temporal distribution of weekly total cases from 2016 to 2019.

Figure 1A represents the weekly number of HAV cases from 2016 to 2019. Time series plots of weekly HAV cases in selected districts, Seoul-si Jongno-gu, Busan-si Sasang-gu, Daejeon-si Seo-gu, and Gyeonggi-do Bucheon-si, are shown in Figure 1B. We found that the temporal distribution of weekly HAV cases varied across districts. Figure 1C compares the temporal variation in the cases from 2016 to 2019. In 2016, the number of HAV cases was high in the spring, but in 2019, it was high in the summer. There was little change in the number of HAV cases in 2018 compared with that of the other years.

**Figure 2:**
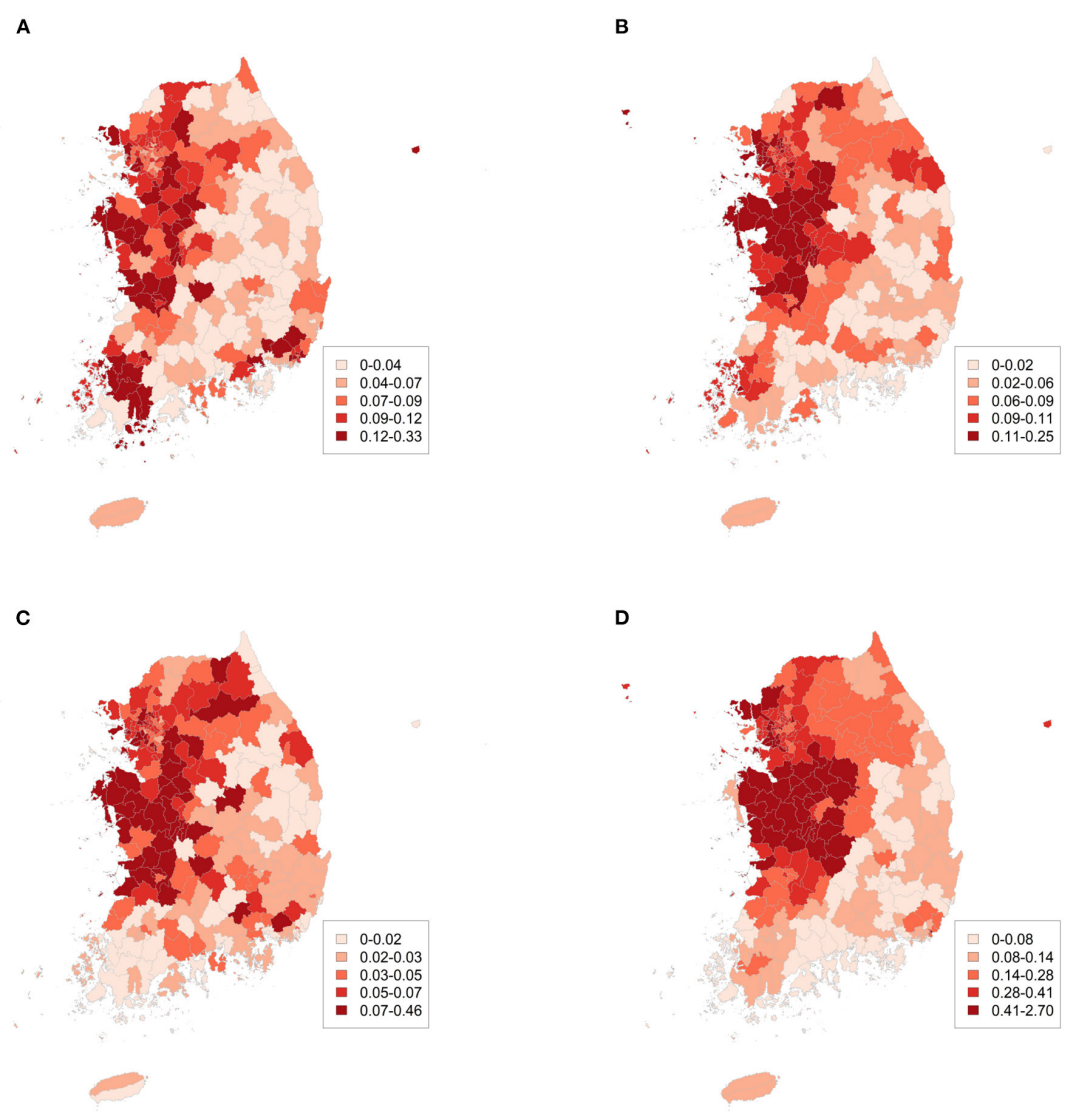
Number of HAV cases per 1,000 people in **(A)** 2016, **(B)** 2017, **(C)** 2018, and **(D)** 2019.

Figures 2A–D illustrate the number of cases per 1,000 people in 2016, 2017, 2018, and 2019, respectively. Overall, the central region of Korea, the Seoul metropolitan area, had a higher number of cases than that in the other regions. The Chungcheong Province, which is close to the Seoul metropolitan region, had a greater number of cases per 1,000 people than that in the other regions.

**Figure 3:**
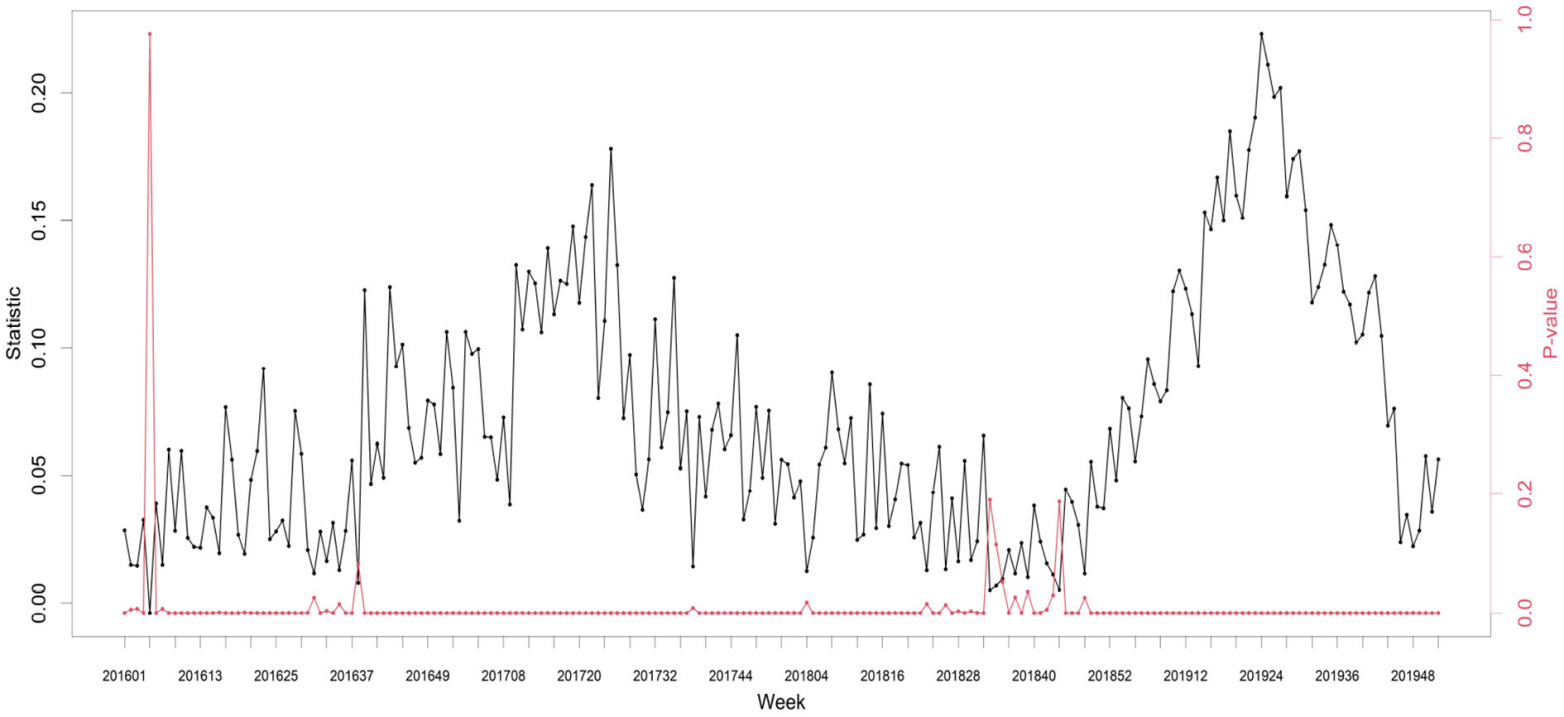
Time series plot of weekly Moran's I and its P-value from 2016 to 2019.

Figure 2 shows the existing connections of observations between the different districts. The more correlated the cases, the closer the districts were to the map. Table 3 presents the global Moran's I values for each year. Here, we calculated the distance-based global Moran's I value and found a positive correlation in space. Figure 3 presents the time series plot of weekly Moran's I and its *P*-values. We also found spatial dependence of HAV cases at most time points.

**Table 3:** Distance-based global Moran's I from 2016 to 2019.

	**2016**	**2017**	**2018**	**2019**
Moran's I	0.138	0.304	0.246	0.186

**Figure 4:**
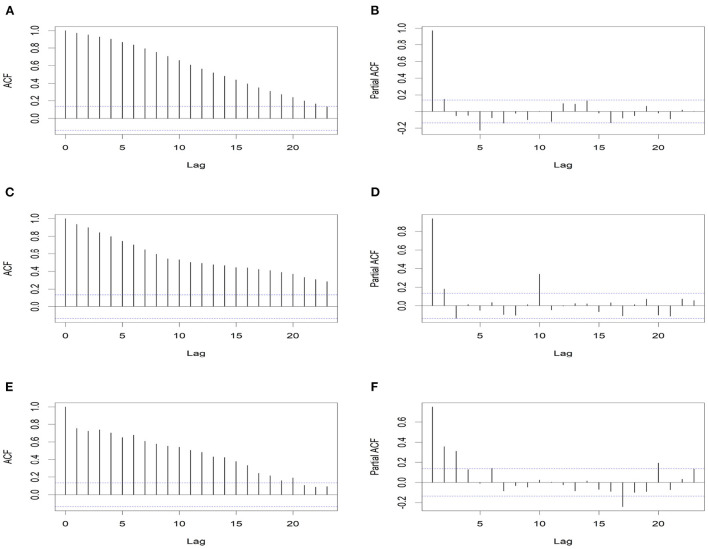
ACF and PACF plots of weekly number of cases. **(A, B)** are for whole districts, **(C, D)** are for Daejeon-si Seo-gu, and **(E, F)** are for Gyeonggi-do Bucheon-si.

Figure 4 shows the ACF and PACF for all districts and each selected district. They are important tools in the exploratory data analysis of time series and, in particular, help to understand the correlation between observations at different time points. It is evident that the time series values at Daejeon-si Seo-gu and Gyeonggi-do Bucheon-si are related to their past values. Thus, considering temporal dependencies when modeling HAV cases is required. Figures 3, 4 show that we must not ignore not only temporal dependence but also spatial dependence. Thus, we included both spatial- and temporal-dependent structures in the model for a better fit.

### 3.2. Bayesian spatio-temporal model

We evaluated the performance of the proposed model, with several competing models as follows: Poisson and ZIP distributions were considered. Type 1 included only fixed factors without a random component. Type 2 included both fixed factors and spatio-temporal random components *ST*_*s,t*_ on the right side of (2). Type 3 also includes fixed factors and spatio-temporal random components within the two-stage framework. The proposed model was a ZIP model with type 3.

Table 4 presents the comparison results of the models, with the mean absolute error (MAE), mean squared prediction Error (MSPE), and deviance information criterion (DIC=Dbar+pD) (46). In general, smaller values of the model fit criterion indicate a better model than that of the competitors. The proposed model (ZIP with type 3) had a smaller MAE and MSPE than that of the other models. Overall, the ZIP models provide slightly better performance than that of the Poisson models in terms of MAE, MSPE, and DIC. The ZIP model with type 2 has a slightly smaller DIC than that of the proposed model. However, the parameter estimation result of a ZIP with the ST model suffered from the spatial confounding bias issue, providing many insignificant coefficients due to the spatio-temporal random components. Thus, we preferred the proposed model in terms of model fit and better interpretation.

**Table 4:** Model comparison results.

**Distribution**	**Type**	**MAE**	**MSPE**	**Dbar**	**DIC**	**pD**
	1	0.745	2.595	128,717	128,727	11
Poisson	2	0.479	0.917	76,911	78,239	1,328
	3	0.366	0.329	66,743	73,175	6,432
	1	0.742	2.583	128,017	128,028	11
ZIP	2	0.465	0.858	72,699	**73,049**	350
	3	**0.365**	**0.328**	66,657	73,119	6,462

The bold values indicate the smallest value of MAE, MSPE, and DIC.

All the variables considered in our proposed model were statistically significant at a significance level of 0.05. The parameter estimates are presented in Table 5. Total income per person, high education rate, total fertility rate, the proportion of males, and the number of foreigners were positively associated with the number of HAV cases. However, the proportion of people aged 30–49 years and the number of doctors per 1,000 people were negatively associated with the number of cases. In addition, we found a negative association between environmental factors, including water supply rate, sewage treatment facility rate, and cases. This indicates that the higher the water quality of the environment, the lower the HAV incidence rate. For weather-related factors, the coefficient of average temperature had a positive value, and the coefficient of precipitation and humidity had a negative value with a small absolute value.

**Table 5:** Parameter estimates from the proposed model.

**Variable type**	**Variable**	**Posterior mean**	**2.5%**	**97.5%**
Socioeconomic factors	Total income per person	0.042	0.039	0.047
	High education rate	0.025	0.019	0.030
	Total fertility rate	0.437	0.332	0.769
	Proportion of males	0.367	0.286	0.408
	Proportion of people with 30–49 years old	–0.138	–0.150	–0.127
	Log(number of foreigners)	0.517	0.493	0.577
	Number of doctors per thousand people	–0.171	–0.183	–0.161
Environmental factors	Water supply rate	–0.028	–0.034	–0.024
	Sewage treatment facility rate	–0.031	–0.034	–0.019
Weather-related factors	Average temperature	0.038	0.036	0.040
	Total precipitation	–0.001	–0.002	–0.001
	Average humidity	–0.006	–0.008	–0.003

## 4. Discussion

We investigated the spatio-temporal distribution of the HAV incidence data in Korea from 2016 to 2019 with visualization methods and various statistical methods such as ACF, PACF, and Moran's I. The results showed that the spatial distribution of HAV incidence varied dynamically over the temporal period of interest and that the temporal distribution varied across districts. We also found that the yearly temporal distribution of HAV cases in Korea is quite different. We found a high peak and significant temporal variation in 2019. Son et al. (47) reported that the ingestion of salted clams significantly increased the risk of HAV in Korea in 2019.

Several HAV studies have been limited to frequency analysis, spatial correlation exploration using Moran's I, and comparisons of SIR and RR. Frequency analysis is useful for finding the frequency of a variable in the entire data, but it is difficult to find a pattern when multiple variables are given conditionally. For example, our frequency analysis in Supplementary Table S2 showed that HAV cases increases up to the 80% quantile of water supply rates and sewage treatment facility rates, and then decreases in the quantile beyond that. However, such results were inconsistent with previous studies (4, 9), and it was known that these factors were related to other socioeconomic factors and had spatial variations. Therefore, frequency analysis alone has a limitation in investigating the association between environmental factors and HAV. Moran's I index is useful for investigating spatial correlation at a fixed time point, but it has a limitation in that it cannot simultaneously determine spatio-temporal correlation. SIR and RR are mainly used to identify patterns of disease occurrence. By representing the SIR and RR values on a map, it is easy to identify the regions at high risk for disease. However, it is difficult to reflect the spatio-temporal correlation using SIR and RR simultaneously. For example, Moon et al. (19) investigated the incidence rates in Korea from 2011 to 2013 by year and age groups and represented RR in specific regions by year. They focused more on frequency analysis and could not consider spatio-temporal dependent structures simultaneously. Examining each variable separately, without considering multiple variables simultaneously, may result in a biased conclusion. In this respect, a regression model with multiple variables is better than that of frequency analysis. Furthermore, it is important to consider spatio-temporal association for epidemic data simultaneously.

Our study proposed spatio-temporal modeling of weekly incidence data in Korea using a Bayesian approach to better explain the complicated spatio-temporal dependence structures of HAV cases. The model assessed the effects of socioeconomic, environmental, and weather factors on weekly HAV cases by adjusting the spatio-temporal dynamics.

The contribution of this study is to examine the spatio-temporal distribution of HAV cases using various exploratory data analysis methods and to develop a Bayesian spatio-temporal model for considering simultaneous spatio-temporal dependent structures of the data. In terms of modeling, our contributions are as follows. Because the onset of infectious diseases has spatial and temporal correlations, a regression model that does not reflect these variations may result in a poor model fit. Considering this point, we applied a regression model with spatio-temporal variations to HAV data in Korea. We attempted to find a model that best reflects spatio-temporal variation. We applied the two-stage framework following (43) to avoid spatial confounding bias issues; thus, we obtained a better model fit than that of the other models. Moreover, the HAV cases are counted and contain many zero values, and we consider the ZIP regression as the base model. Using the ZIP regression model coupled with two-stage and spatio-temporal structures, we demonstrated that spatio-temporal variation could not be neglected in analyzing an epidemic disease.

In the proposed spatio-temporal model, various socioeconomic, environmental, and weather-related factors were statistically significant for HAV occurrence in Korea from 2016 to 2019. The results showed that the higher the level of income and education, the more social activities, and the more frequent contact with people, the higher the possibility of exposure to HAV in Korea. It also showed that the higher the male ratio and number of foreigners residing, the higher the HAV incidence rate. Our findings agree with the studies mentioned earlier in Korea (19, 25, 35). Moreover, the number of medical doctors was negatively associated with the HAV incidence rate, as mentioned by Choi (35). We found that the proportion of people aged 30–49 years and incidence had a negative association after adjusting various socioeconomic and environmental factors. This result is somewhat inconsistent with the previous study of Yoon et al. (6). There might be confounding factors that were not considered in our study. Explanations for the present findings warrant further study on the association of the proportion of people with specific age groups and HAV.

We found that the coefficients of the water supply and sewage treatment facility rates were negative, indicating that the higher the water quality and hygiene conditions, the lower the incidence rate of HAV. These results are in line with previous studies (8, 9), even though our exploratory data analysis in Supplementary Table S2 looked like a positive association. Thus, we again confirmed the importance of spatio-temporal multiple regression modeling to examine the association between factors and HAV simultaneously.

For weather-related factors, the coefficient of average temperature had a positive value, and the coefficient of precipitation and humidity had a negative value with a small absolute value. Supplementary Figure S3 showed the positive association between average temperature and HAV cases, although the associations between other weather factors and HAV were not clearly shown. Moreover, Supplementary Table S3 indicated that the number of HAV cases was relatively large in the spring and summer seasons. A clear seasonal variation was observed in 2019. In Korea, the incidence of HAV is relatively high during spring and summer because of increased outdoor activity and ingestion of not clean food handling (19). Thus, we conclude that temperature is more associated with HAV outbreaks than precipitation or humidity in Korea.

As in the existing studies on HAV in Korea (19, 25, 35), we confirmed that there are risk factors for HAV occurrence. The distribution status of HAV varies by region and time. Additionally, differences in socioeconomic variables, such as education level, sex, number of medical doctors, and water quality, affect the number of HAV cases. Environmental and weather-related factors are also important; however, we found that the contribution of socioeconomic factors is more crucial for HAV occurrence. Therefore, we should recognize the different factors in different regions and prepare region-specific control and prevention strategies for HAV infection. Furthermore, Kang et al. (26) mentioned that a particular age group has a low antibody cultivation rate and is more vulnerable to infection. Therefore, we must consider an age-specific strategic vaccine plan.

Association between socioeconomic factors and HAV prevalence may vary from region to region because the different areas have different characteristics. For example, Jacobsen and Koopman (48) described that a higher level of education leads to a sustained decrease in the incidence of HAV, whereas there was no statistically significant difference in education when examining HAV antibodies between sewage workers in France and the control group in Cadilhac and Roudot-Thoraval (49). While Rachiotis et al. (50) showed that people with higher education levels had higher rates of anti-HAV in stratified analysis among municipal waste collectors, Arvanitidou et al. (51) showed that the prevalence of anti-HAV was significantly higher in less educated persons. Our exploratory data analysis (Supplementary Table S2) and modeling results provided the positive association between higher education rate and HAV in Korea during 2016–2019. In Korea, people with higher education background tend to have more active social lives and more frequent contact with people so they may have highly exposure to HAV. Seo et al. (25) reported a similar result in Korea. Thus, it is important to consider regional characteristics along with weather-related factors to better understand HAV across Korea.

There was a limitation concerning the data in this study. We focused on regional aggregated data, which could lead to biased results. Thus, ascertaining the direct relationship between factors and outcomes can be limited. If we obtain individual-level HAV case data with individual-level risk factors and conduct spatio-temporal data analyzes, we can find more features that influence the HAV cases and draw clearer pictures of the infection spread problem. Thus, this is one of the future research directions the authors intend to pursue.

## Data availability statement

The number of weekly HAV cases can be found in the Korea Disease Control and Prevention Agency database (https://www.kdca.go.kr/index.es?sid=a3). The socioeconomic and environmental datasets can be found in the Korean Statistical Information Service (https://kosis.kr/eng/) and Statistics Korea (https://kostat.go.kr/portal/eng). The weather-related datasets were obtained from the Korea Meteorological Administration (http://www.kma.go.kr/eng/index.jsp).

## Author contributions

JJ and JC performed the statistical analyzes. All authors contributed to the conception and design of the study, organized the database, and wrote the manuscript. All authors contributed to the article and approved the submitted version.
