# Supplementary material for: Investigating the spatio-temporal variation of hepatitis A in Korea using a Bayesian model
Source: Front Public Health. 2023 Jan 20;10:1085077. doi: 10.3389/fpubh.2022.1085077 (PMC9895396; doi:10.3389/fpubh.2022.1085077)
Supplement: Supplementary file 1 [file Data_Sheet_1.pdf]

Supplementary Material

1 DESCRIPTION OF DATA

Table S1 reported the data source for the outcome and factors in this study. For the socioeconomic and environmental factors, we obtained datasets from different statistics from various institutions. However, all statistics can be accessed through the Korean Statistical Information Service (KOSIS). For the weather-related factors, we obtained observations from two systems, an automatic weather system (AWS) and an automated synoptic observing system (ASOS), and then combined them and performed Kriging to form datasets.

Table S1. Data source of data set (outcome, socioeconomic factors, environmental factors).

Variable type	Variable	Data source
Outcome	Hepatitis A cases	Korea Disease Control and Prevention Agency
Socioeconomic factor	total income per person (one million won)	National Tax Service
	high education rate (%)	Statistics Korea
	total fertility rate per woman of 15-49 years old	Statistics Korea
	proportion of males (%)	Statistics Korea
	proportion of people with 30-49 years old (%)	Statistics Korea
	log(number of foreigners)	Ministry of the Interior and Safety
	number of doctors per thousand people	Health Insurance Review & Assessment Service
Environmental factor	water supply rate (%)	Ministry of Environment
	sewage treatment facility rate (%)	Ministry of Environment
Weather-related factor	average temperature (°C)	Korea Meteorological Administration
	total precipitation (mm)	Korea Meteorological Administration
	average humidity (%)	Korea Meteorological Administration

2 EXPLANATORY DATA ANALYSIS

2.1 Socioeconomic and environmental factors

Figure S1 presented the spatial distribution of socioeconomic and environmental factors. The average value for each factor was used from 2016 to 2019. Table S2 reported the number of HAV cases per 1,000 by the group for each variable in 2016-2019. Five groups were categorized based on quantile information. Figure S2 presented the scatterplots of socioeconomic and environmental factors versus the corresponding number of HAV cases per 1,000 people in 2016-2019.

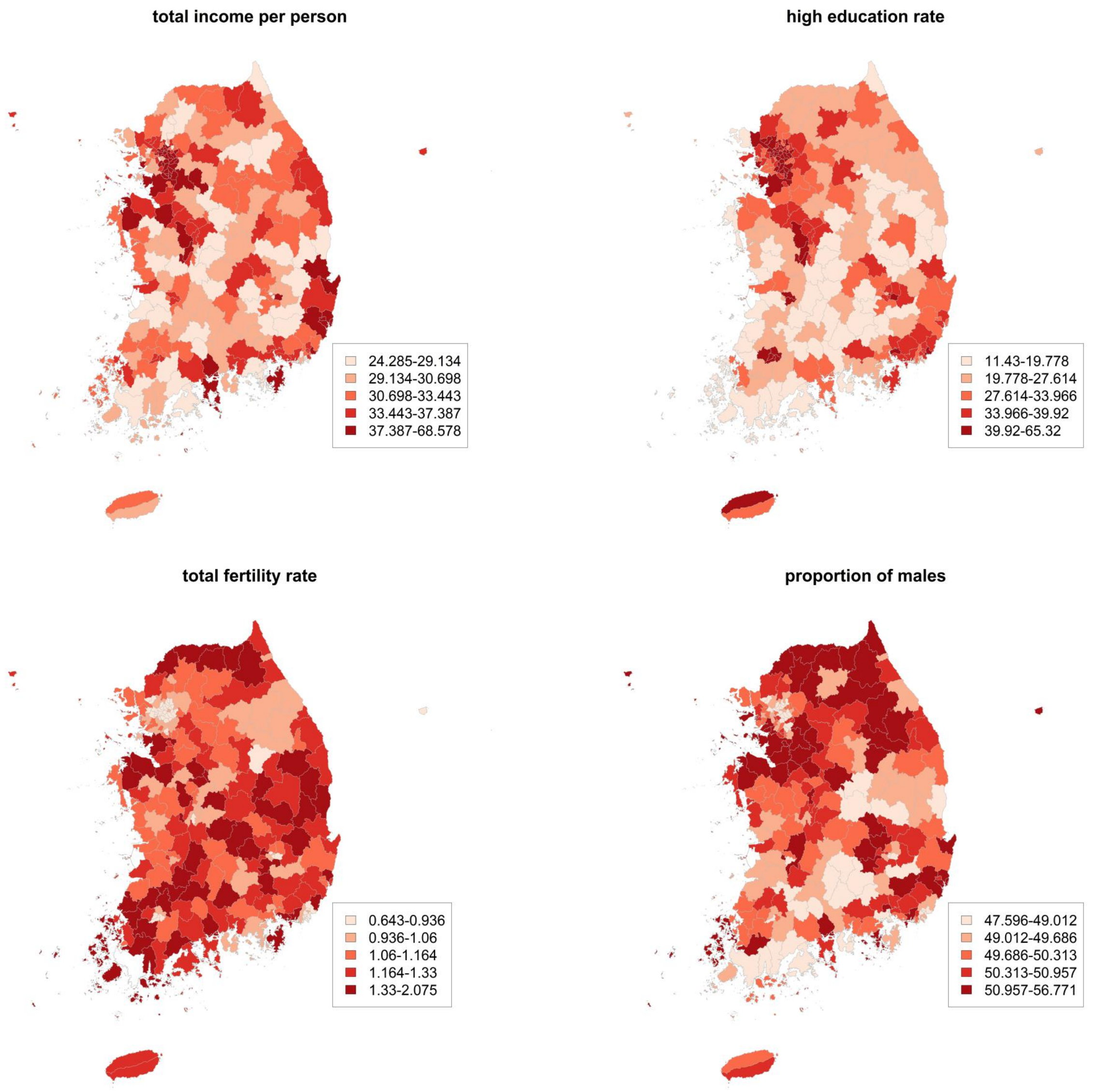

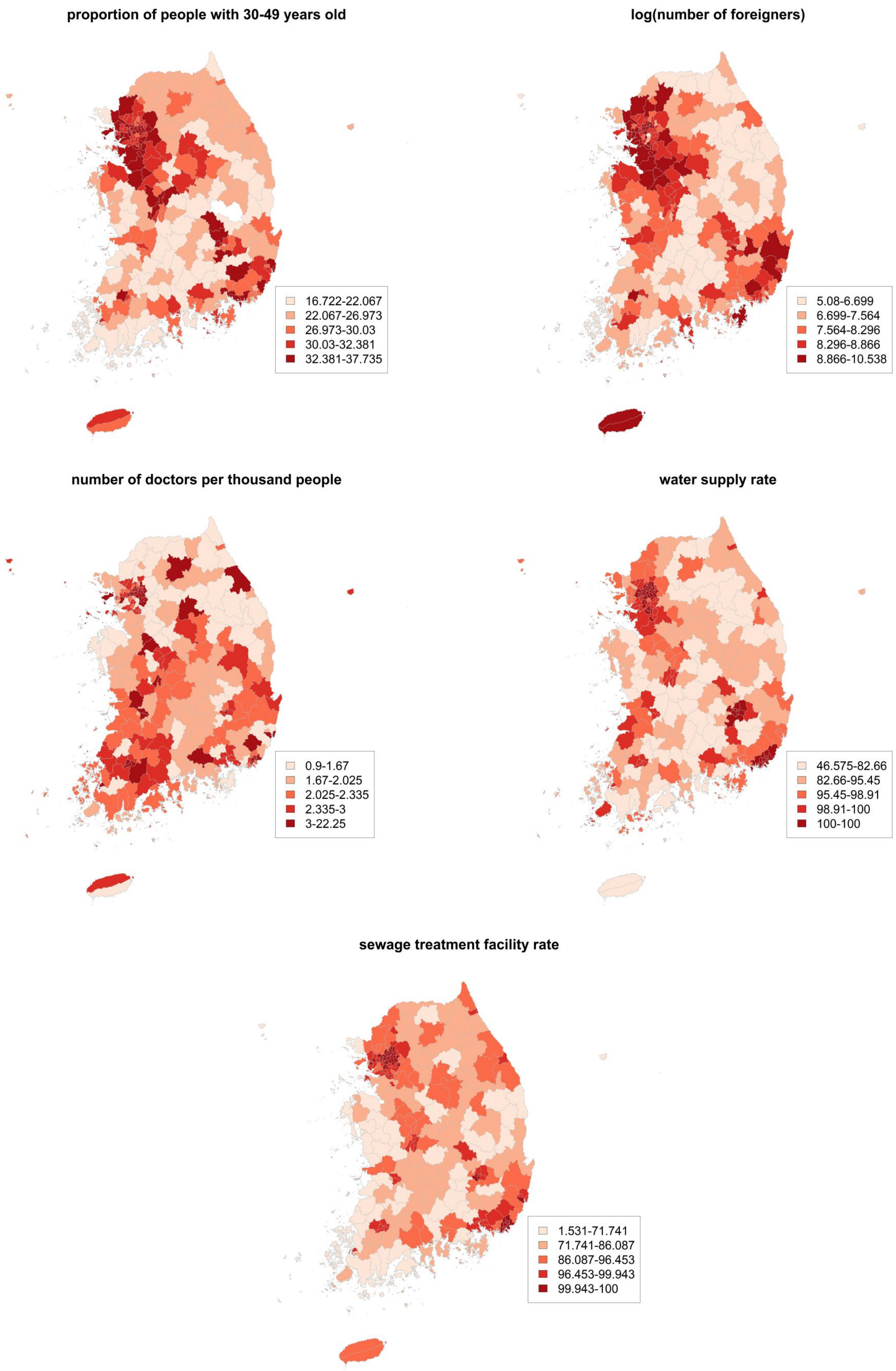

Figure S1: Spatial distribution of socioeconomic and environmental factors

Table S2. Number of HAV cases per 1,000 people by the group for each variable. G1, G2, G3, G4 and G5 are corresponding to 0–20%, 20–40%, 40–60%, 60–80% and 80–100% quantiles, respectively.

Variable	Group	Range	Min	Q1	Median	Mean	Q3	Max
total income per person	G1	24.28 - 29.13	0.036	0.162	0.405	0.595	0.571	2.936
	G2	29.13 - 30.70	0.000	0.181	0.299	0.528	0.646	3.154
	G3	30.70 - 33.44	0.034	0.255	0.405	0.506	0.650	2.613
	G4	33.44 - 37.39	0.066	0.202	0.363	0.481	0.570	2.402
	G5	37.39 - 68.58	0.115	0.442	0.574	0.578	0.655	1.681
high education rate	G1	11.43 - 19.78	0.000	0.128	0.244	0.470	0.520	2.936
	G2	19.78 - 27.61	0.066	0.192	0.310	0.507	0.505	3.154
	G3	27.61 - 33.97	0.083	0.194	0.491	0.567	0.772	2.530
	G4	33.97 - 39.92	0.115	0.245	0.532	0.531	0.656	2.613
	G5	39.92 - 65.32	0.078	0.465	0.557	0.615	0.638	2.402
total fertility rate	G1	0.64 - 0.94	0.062	0.300	0.510	0.503	0.611	2.613
	G2	0.94 - 1.06	0.083	0.265	0.491	0.625	0.645	2.936
	G3	1.06 - 1.16	0.025	0.253	0.558	0.558	0.763	1.617
	G4	1.16 - 1.33	0.000	0.180	0.282	0.492	0.566	2.790
	G5	1.33 - 2.08	0.038	0.161	0.255	0.512	0.492	3.154
proportion of males	G1	47.60 - 49.01	0.025	0.186	0.331	0.363	0.545	0.739
	G2	49.01 - 49.69	0.115	0.199	0.309	0.483	0.574	2.613
	G3	49.69 - 50.31	0.066	0.232	0.532	0.602	0.737	2.359
	G4	50.31 - 50.96	0.000	0.209	0.526	0.718	0.794	2.936
	G5	50.96 - 56.78	0.062	0.213	0.430	0.525	0.689	3.154
proportion of people aged 30-49 years	G1	16.72 - 22.07	0.000	0.138	0.250	0.498	0.567	2.790
	G2	22.07 26.97	0.100	0.189	0.277	0.452	0.477	2.936
	G3	26.97 - 30.03	0.062	0.197	0.347	0.570	0.590	3.154
	G4	30.03 - 32.38	0.097	0.476	0.572	0.587	0.657	2.402
	G5	32.38 - 37.74	0.078	0.441	0.565	0.583	0.690	1.681
log(number of foreigners)	G1	5.08 - 6.70	0.000	0.132	0.290	0.457	0.488	2.936
	G2	6.70 - 7.56	0.093	0.184	0.250	0.360	0.379	2.613
	G3	7.56 - 8.30	0.066	0.193	0.402	0.558	0.600	2.790
	G4	8.30 - 8.87	0.154	0.392	0.547	0.671	0.761	3.154
	G5	8.87 - 10.54	0.115	0.523	0.621	0.645	0.711	2.759
number of doctors per thousand people	G1	0.90 - 1.67	0.000	0.201	0.405	0.574	0.618	3.154
	G2	1.67 - 2.03	0.038	0.159	0.351	0.448	0.708	1.223
	G3	2.03 - 2.34	0.034	0.182	0.304	0.642	0.639	2.936
	G4	2.34 - 3.00	0.036	0.243	0.499	0.476	0.634	1.252
	G5	3.00 - 22.25	0.128	0.307	0.508	0.545	0.623	2.613
water supply rate	G1	47.60 - 82.66	0.000	0.155	0.255	0.453	0.463	2.790
	G2	82.66 - 95.45	0.038	0.196	0.293	0.597	0.727	3.154
	G3	95.45 - 98.91	0.066	0.216	0.523	0.514	0.660	1.625
	G4	98.91- 100.00	0.127	0.303	0.544	0.724	0.776	2.613
	G5	100.00 - 100.00	0.128	0.245	0.490	0.448	0.615	0.876
sewage treatment facility rate	G1	1.53 - 71.74	0.000	0.170	0.286	0.498	0.618	2.759
	G2	71.74 - 86.09	0.025	0.161	0.282	0.503	0.492	3.154
	G3	86.09 - 96.45	0.036	0.245	0.532	0.626	0.790	2.936
	G4	96.45 - 99.94	0.078	0.226	0.542	0.608	0.645	2.613
	G5	99.94 - 100.00	0.128	0.289	0.499	0.456	0.609	0.739

2.2 Weather-related factors

Table S3 reported the weekly average number of HAV cases by season and year. Figure S3 presented the scatterplots of weekly weather variables versus the corresponding number of HAV cases in 2016–2019.

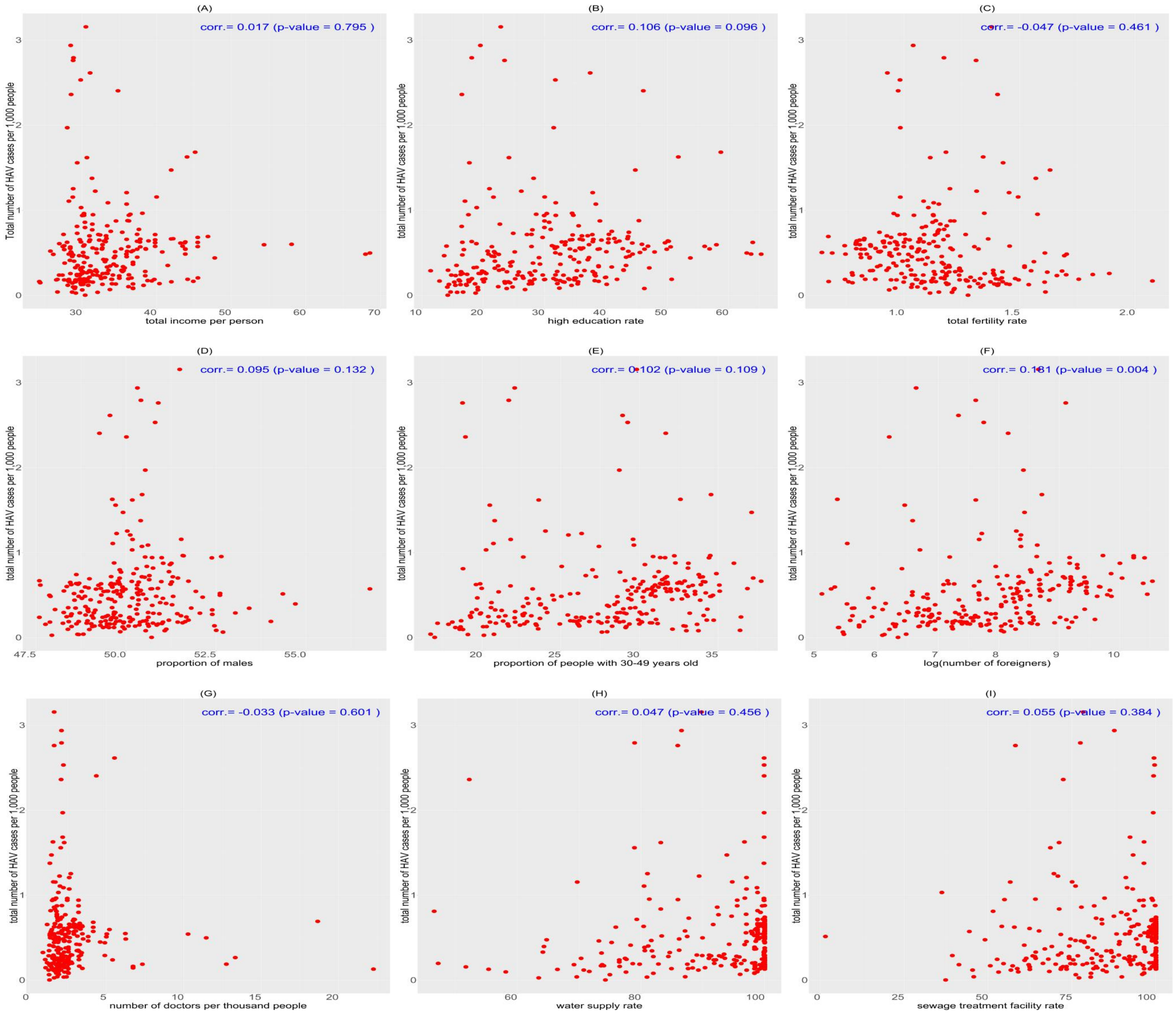

Figure S2: Scatterplots of socioeconomic and environmental factors versus the corresponding number of HAV cases per 1,000 people in 2016–2019

Table S3. Weekly averaged number of HAV cases by season and year

Season	2016	2017	2018	2019
Winter I (Weeks 1 to 9)	54	95	66	118
Spring (Weeks 10 to 22)	148	114	56	401
Summer (Weeks 23 to 35)	68	82	34	574
Fall (Weeks 37 to 49)	71	62	35	275
Winter II (Weeks 50 to 53)	95	54	47	67

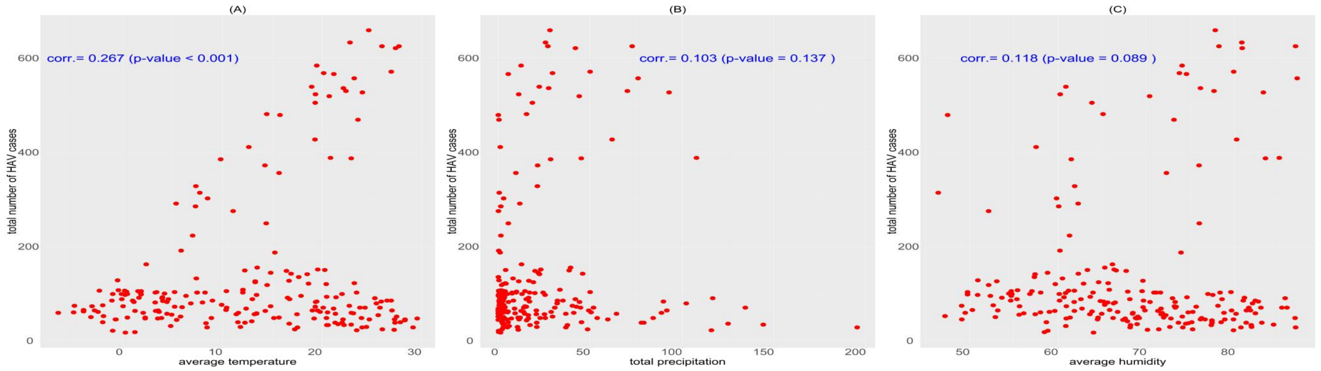

Figure S3: Scatterplots of (A) average weekly temperature, (B) total weekly precipitation, and (C) average weekly humidity versus the corresponding number of HAV cases in 2016–2019
